# Next-Generation Bionic Sensors for Small Molecule Detection: Integrating Synthetic Biology, Nanomaterials, and Artificial Intelligence

**DOI:** 10.3390/mi17060725

**Published:** 2026-06-15

**Authors:** Yasmin Barazandegan, Dipsana Kc, Rebecca Iha, Niya Tu, Nadia Ryan, Pietro Martano, Xavier Jones, John Yang, Ruipu Mu, Qingbo Yang

**Affiliations:** 1 Cooperative Research, College of Agriculture, Environmental and Human Sciences, Lincoln University of Missouri, Jefferson City, MO 65101, USAdipsana.k.c.687@my.lincolnu.edu (D.K.);; 2 The Basic Sciences Department, University of Health Sciences and Pharmacy in St. Louis, St. Louis, MO 63110, USA

**Keywords:** bionic sensors, small molecule detection, nanomaterials, artificial intelligence, biosensing in complex matrices, wearable biosensors

## Abstract

Bionic sensors are emerging as powerful analytical platforms driving the development of next-generation detection technologies, particularly for small molecule sensing in complex environmental and biological systems. However, accurate and selective detection of small molecules remains fundamentally challenging due to their low molecular weight, limited structural specificity, and strong interference from complex matrices. This review provides a comprehensive overview of recent advances in bionic sensor technologies, focusing on how the integration of synthetic biology, nanomaterials, and artificial intelligence (AI) addresses these limitations. Key biorecognition elements, including enzymes, antibodies, aptamers, and molecularly imprinted polymers, are examined for their suitability in small molecule sensing applications. Advances in nanomaterials such as graphene, carbon nanotubes, quantum dots, and MXenes are discussed in relation to signal transduction enhancement, sensitivity improvement, and device miniaturization. In parallel, the roles of AI and machine learning in signal denoising, adaptive calibration, and molecular fingerprinting for complex datasets are highlighted. Applications in wearable and implantable biosensors, environmental monitoring, and food safety are analyzed, emphasizing real-time detection of metabolites, pollutants, and toxins. Key challenges associated with AI-driven systems, including scalability, cost, data reliability, and ethical concerns, are also discussed. Emerging trends such as hybrid sensing platforms, self-powered biosensors, and secure data integration frameworks are presented as future directions. This review aims to provide a problem-driven perspective on how next-generation bionic sensors can overcome current limitations and enable robust small molecule detection in real-world applications.

## 1. Introduction to Bionic Sensor Technologies

### 1.1. Foundations

Biosensors are innovative analytical devices that detect biological signals and convert them into measurable outputs, such as electrical, optical, or electrochemical signals [1]. Their ability to provide fast, specific, and cost-effective detection has made them essential tools in healthcare, food safety, environmental monitoring, and more [2]. A typical biosensor includes three main parts: a bioreceptor that recognizes the target molecule, a transducer that converts the biological response into a signal, and a system to amplify and process that signal for analysis [3]. To fully appreciate the capabilities of today’s biosensors, it’s helpful to understand how they evolved.

### 1.2. Brief History and Evolution of Bionic Sensing Mechanisms

**Figure 1:**
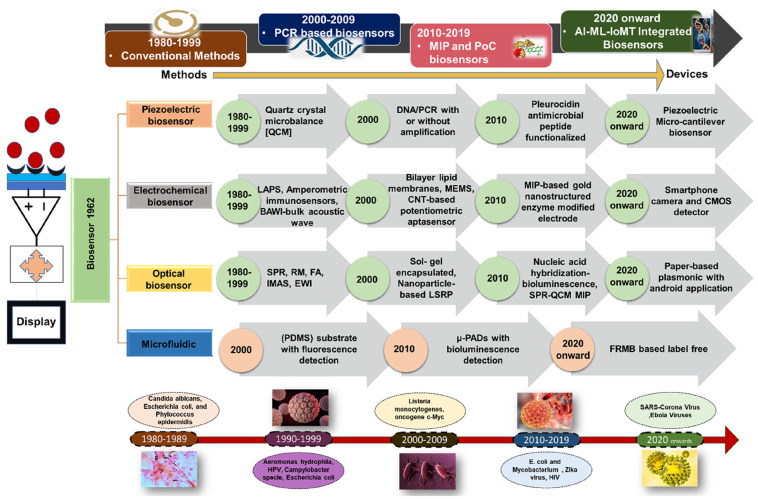
Evolution of biosensor technologies from conventional sensing platforms to AI-integrated next-generation biosensors. The schematic illustrates major advances in electrochemical, optical, piezoelectric, and microfluidic biosensing systems, including the integration of nanomaterials, molecular recognition elements, and artificial intelligence for biomedical, environmental, and food-related applications. Adapted from [13], Bioengineering & Translational Medicine, under the terms of the CC BY license.

The journey of biosensors began in the 1960s with the groundbreaking work of Clark and Lyons, who developed the first enzyme-based sensor for glucose detection [4,5] This innovation was soon followed by Updike and Hicks (1967) [6], who introduced enzyme immobilization techniques to improve sensor stability [7]. Over the following decades, biosensors expanded in complexity and purpose. Microbial biosensors utilized whole cells for detection [8,9], while tissue-based sensors relied on plant or animal tissues to identify target substances [10]. Later developments introduced organelle-based biosensors using parts of cells like membranes and mitochondria to enhance selectivity [11]. These early biosensors laid the foundation for more advanced devices, although they were often limited by longer response times and lower precision. As the field progressed, immunosensors, DNA biosensors, and piezoelectric biosensors emerged, each offering enhanced speed, accuracy, and sensitivity [12]. Today’s state-of-the-art biosensors have achieved remarkable performance through the integration of nanotechnology, synthetic biology, and artificial intelligence (AI). These combined technologies have created powerful tools for real-time monitoring, automation, and ultra-sensitive detection across diverse sectors. The evolution of biosensor technologies from conventional electrochemical systems to AI-integrated platforms is illustrated schematically in Figure 1, adapted from recent advances summarized by Parihar et al. [13].

### 1.3. Current Technologies and Applications of Bionic Sensors

Modern biosensors are employed across numerous fields due to their adaptability, and high efficiency. Although many of these biosensing platforms were initially developed for the detection of proteins, pathogens, and cellular biomarkers, their relevance to this review lies in their increasing adaptation for small molecule detection, particularly for metabolites, toxins, pollutants, pharmaceutical compounds, and food contaminants. The commercialization of these technologies has increased their accessibility, enabling widespread applications in workplace health monitoring, point-of-care diagnostics, and environmental exposure assessment [14]. As illustrated in Table 1, these sensors can be broadly categorized into three types based on their placement and function: placeable, wearable, and implantable devices each designed for specific operational settings and user needs.

### 1.4. Types of Bionic Biosensors Based on Application Setting

**Wearable biosensors** are non-invasive devices integrated into accessories, patches, or clothing, enabling real-time monitoring of vital signs such as heart rate, glucose levels, and body temperature [16,17]. They are widely used in chronic disease management, workplace safety, fitness tracking, and preventive healthcare applications [15].

**Placeable biosensors** are portable or semi-fixed devices designed for use in external environments or on surfaces. They are commonly deployed in air and water quality monitoring systems to detect pollutants, toxins, and pathogens [14,18]. These biosensors are essential tools in smart packaging, environmental surveillance, and on-site food safety analysis.

**Implantable biosensors** are designed to be embedded within the human body to enable continuous, long-term internal monitoring [20,21]. These devices are widely applied in precision medicine for tracking biomarkers such as glucose concentrations, cardiac enzymes, and neural activity [19]. Their ability to deliver real-time physiological data from within the body makes them indispensable for personalized therapy, chronic disease management, and advanced diagnostics.

In summary, despite the aforementioned advances, achieving accurate and selective detection of small molecules in complex biological and environmental matrices remains a fundamental and long-standing challenge. It is precisely this challenge that has driven the development of the next-generation bionic sensing systems discussed in this review.

### 1.5. Physicochemical Challenges in Small Molecule Detection

Small molecule detection remains one of the most challenging areas in biosensor development due to the intrinsic physicochemical properties of these analytes. Unlike proteins, nucleic acids, cells, or pathogens, small molecules typically possess low molecular weight, simple chemical structures, limited functional groups, and minimal surface epitopes available for molecular recognition. As a result, they often exhibit weak binding interactions with conventional bioreceptors, making highly selective and sensitive detection difficult, particularly in complex biological and environmental samples [22,23]. In addition, many small molecules share highly similar structural and electrochemical characteristics with interfering compounds, increasing the risk of cross-reactivity, signal overlap, and false-positive responses.

The analytical complexity of small molecule sensing is further intensified by matrix interference, nonspecific adsorption, biofouling, and low target abundance in real-world samples. Biological fluids, food matrices, and environmental samples often contain proteins, salts, metabolites, lipids, and particulate contaminants that interfere with analyte recognition and signal transduction [24,25]. Furthermore, the rapid diffusion behavior and transient interactions of small molecules can reduce residence time at sensing interfaces, thereby limiting detection sensitivity and reproducibility. These challenges are particularly problematic in wearable, implantable, and point-of-care biosensors that require continuous real-time monitoring under dynamic physiological conditions [26,27].

To address these limitations, advanced nanomaterials such as graphene, carbon nanotubes (CNTs), quantum dots, and MXenes have been increasingly integrated into biosensor platforms to improve molecular recognition and signal amplification. Their large surface area, tunable surface chemistry, high electrical conductivity, and enhanced electron transfer capabilities enable more efficient immobilization of bioreceptors and stronger signal generation even at ultra-low analyte concentrations [28,29]. In particular, graphene- and CNT-based nanocomposites improve sensitivity by facilitating rapid charge transfer, while MXenes provide highly modifiable surfaces that enhance selectivity and electrochemical responsiveness in complex sensing environments.

Artificial intelligence (AI) and deep learning algorithms are also playing an increasingly important role in overcoming the limitations of small molecule detection. Machine learning models, including convolutional neural networks (CNNs), deep neural networks (DNNs), and adaptive calibration systems, can distinguish weak analytical signals from background noise, compensate for signal drift, and identify complex molecular fingerprints within multidimensional datasets [30,31]. These computational approaches significantly improve detection accuracy, particularly in cases involving overlapping spectral features, low analyte abundance, and matrix-induced interference. AI-assisted signal denoising and pattern recognition therefore represent critical advances toward robust real-time biosensing in practical applications.

Collectively, these challenges have driven the development of next-generation bionic sensing systems that combine advanced biorecognition strategies, nanomaterial engineering, and artificial intelligence to achieve improved sensitivity, selectivity, and real-world applicability for small molecule detection.

## 2. Biological and Synthetic Recognition Elements in Bionic Sensors

Bionic sensors utilize engineered biological and synthetic recognition elements, also known as bioreceptors, to detect target molecules with high precision and specificity. These bioreceptors are essential for enabling real-time identification of analytes, supporting critical applications in medical diagnostics, environmental surveillance, food safety, and the pharmaceutical industry [32,33,34]. The choice of bioreceptor significantly influences the biosensor’s performance in terms of specificity, stability, and scalability [35]. Among these, the selection of appropriate bioreceptors is particularly critical for small molecule detection, as weak binding affinities and limited structural complexity often pose significant challenges to achieving high specificity.

This section provides an overview of commonly used bioreceptors including antibodies, enzymes, aptamers, and molecularly imprinted polymers. It highlights recent innovations in synthetic receptor technologies and examines key challenges that limit their widespread adoption.

### 2.1. Overview of Bioreceptors

Bioreceptors serve as the fundamental recognition units in biosensors, enabling selective binding to specific target molecules. Based on their structural characteristics and recognition mechanisms, biosensors are commonly categorized into immunological, enzymatic, nucleic acid-based, and synthetic receptor-based types (Table 2) [22].

#### 2.1.1. Antibodies

**Figure 2:**
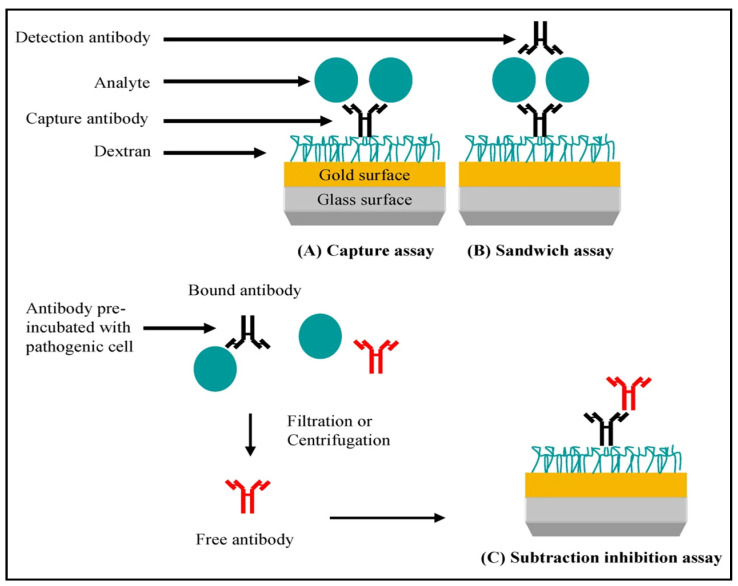
Schematic representation of antibody-based immunoassay formats used in biosensors, including (**A**) capture assay, (**B**) sandwich assay, and (**C**) subtraction inhibition assay for antigen detection. Reproduced from [36], Sensors, under the terms of the Creative Commons Attribution (CC BY) license.

Antibodies are extensively utilized in biosensing applications due to their exceptional specificity toward target antigens. Immunosensors that employ monoclonal or polyclonal antibodies have demonstrated high efficacy in clinical diagnostics, pathogen identification, and environmental surveillance. Common immunosensing formats include capture, sandwich, and inhibition-based assays (Figure 2), adapted from classical immunosensor configurations described by Byrne et al. [36]. Despite their strong binding affinity, performance can be influenced by external factors such as pH, temperature, and ionic strength [37,38].

#### 2.1.2. Enzymes

**Figure 3:**
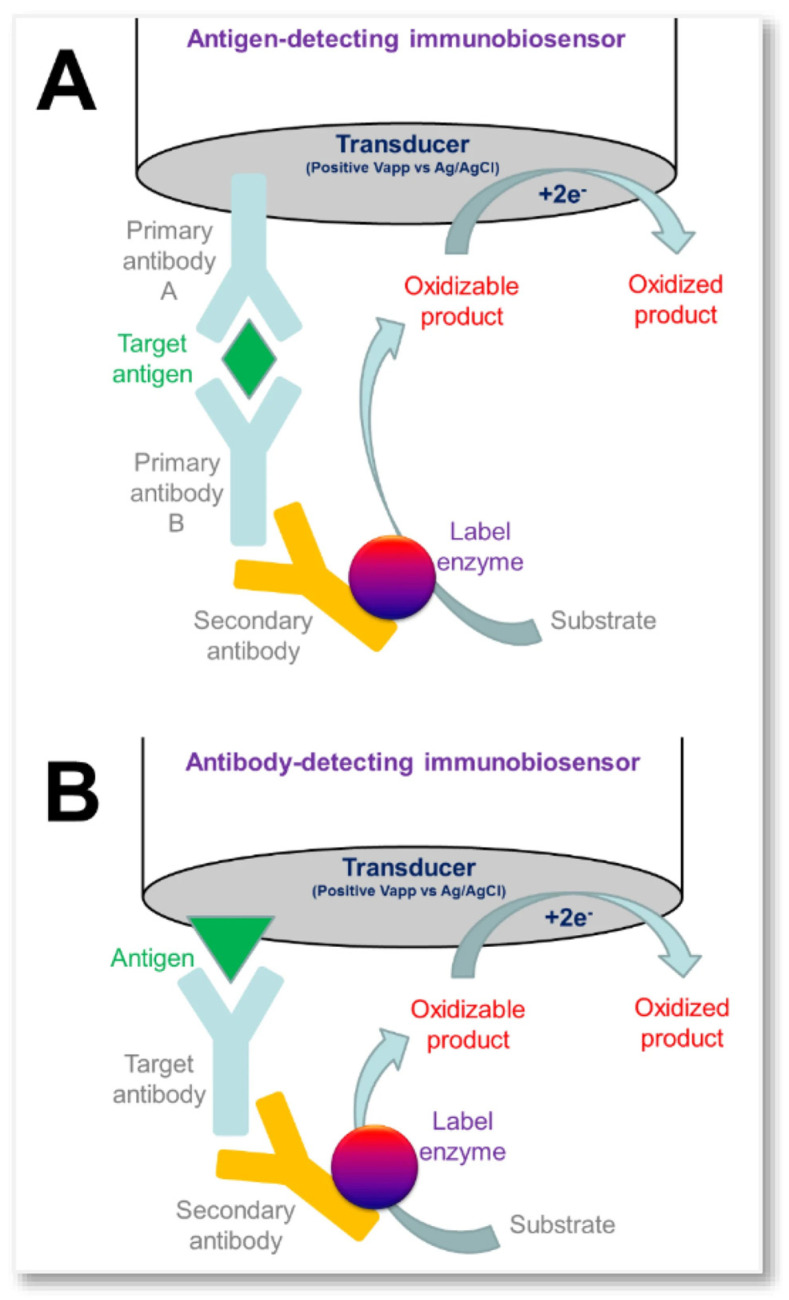
Enzyme-labeled immunobiosensors. (**A**) Schematic representation of an antigen-detecting immunobiosensor. (**B**) Schematic representation of an antibody-detecting immunobiosensor. The enzyme label catalyzes substrate conversion to generate an oxidizable product, which is subsequently detected by the electrochemical transducer. Adapted from [40], Sensors, under the terms of the Creative Commons Attribution (CC BY) license.

Enzymes offer catalytic efficiency and molecular specificity, enabling both quantitative and qualitative detection of analytes. They are frequently employed in biosensors for applications in biomedical diagnostics, agriculture, and food safety [39]. In many biosensing platforms, enzymes function as catalytic labels that generate measurable electrochemical signals through substrate conversion, thereby enabling signal amplification and sensitive detection, as illustrated in Figure 3 [40]. However, enzyme-based sensors often face challenges related to limited operational stability due to their vulnerability to denaturation and degradation over time [41].

#### 2.1.3. Aptamers

**Figure 4:**
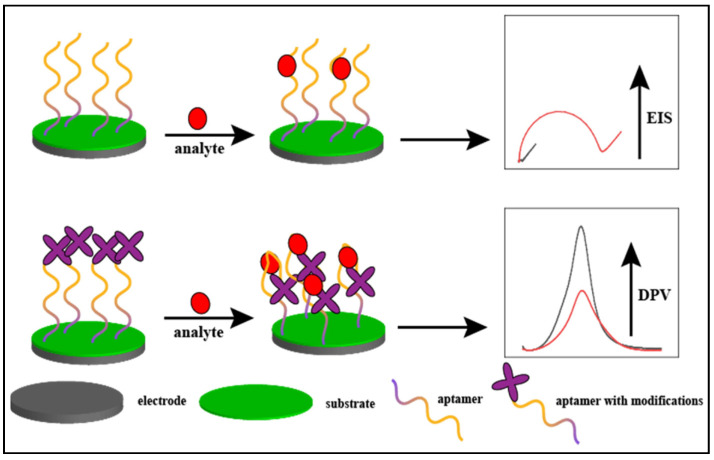
Schematic illustration of an electrochemical aptamer-based biosensor. Binding of the target analyte to surface-immobilized aptamers alters the electrochemical response, which can be detected using techniques such as electrochemical impedance spectroscopy (EIS) and differential pulse voltammetry (DPV). Reproduced from [45], Biosensors, used under the terms of the Creative Commons Attribution (CC BY) license.

Aptamers are synthetic single-stranded DNA or RNA molecules that adopt specific three-dimensional conformations capable of binding target analytes with high affinity and selectivity [42]. Their major advantages include chemical stability, cost-effective synthesis, and adaptability toward a broad range of analytes, including non-immunogenic targets [43]. Aptamer-based biosensors have shown strong potential in microbial detection [44], therapeutic drug monitoring, and clinical diagnostics. A typical mechanism of electrochemical aptamer-based biosensing, in which target binding induces measurable changes in impedance or current signals, is illustrated in Figure 4 [45].

#### 2.1.4. Molecularly Imprinted Polymers (MIPs)

**Figure 5:**
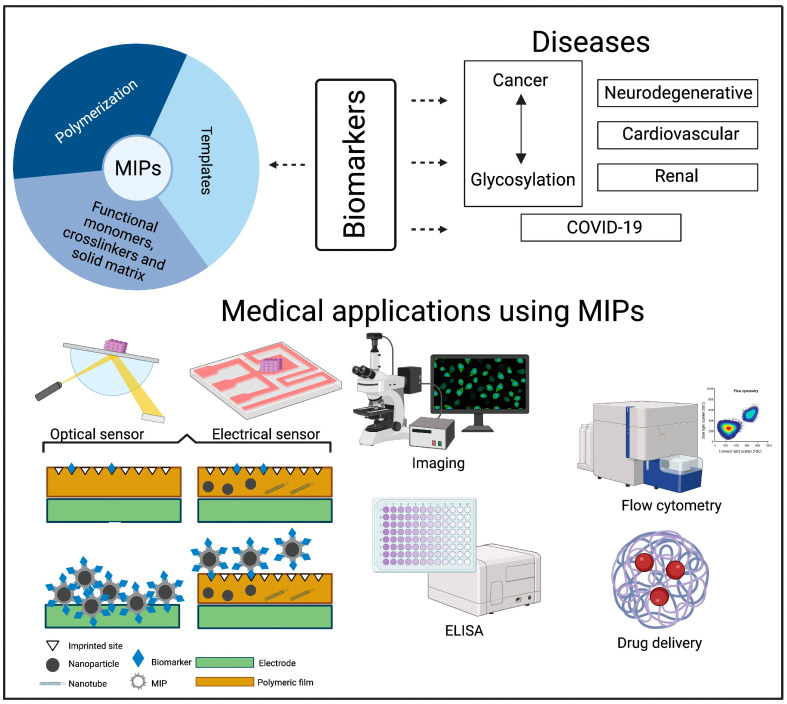
Overview of molecularly imprinted polymers (MIPs) including their fabrication process, biomarker recognition mechanism, and applications in medical diagnostics such as optical and electrical biosensors, imaging, ELISA, flow cytometry, and drug delivery. Adapted from [47], Polymers, used under the terms of the Creative Commons Attribution (CC BY) license.

Molecularly imprinted polymers (MIPs) are synthetic polymeric materials engineered with selective cavities that mimic natural binding sites. These cavities are designed to match the size, shape, and functional groups of a target analyte [23]. Due to their robustness, low production cost, and resistance to harsh environmental conditions, MIPs are particularly attractive for small-molecule detection in industrial, environmental, and biomedical applications [46]. The general design principles of MIPs and their diverse applications in biosensing and medical diagnostics are summarized in Figure 5, adapted from [47].

### 2.2. Advances in Synthetic and Hybrid Recognition Systems

Recent developments in synthetic and hybrid bioreceptors have greatly enhanced biosensor performance, improving their selectivity, sensitivity, and resilience in complex environments.

#### 2.2.1. Nanomaterial Integration

Nanomaterials such as carbon nanotubes, graphene, and quantum dots are increasingly used to improve signal transduction, conductivity, and detection limits. These advances enable biosensors to detect ultra-low concentrations of small molecules with greater speed and accuracy [48].

#### 2.2.2. Smartphone-Compatible Platforms

The integration of biosensors with smartphones has created user-friendly, portable systems for point-of-care testing. These platforms utilize optical or electrochemical readouts, enabling applications in food testing, clinical diagnostics, and environmental surveillance [49].

#### 2.2.3. Hybrid Recognition Elements

New biosensors combine natural receptors (e.g., enzymes, antibodies) with synthetic ones (e.g., aptamers, MIPs) to create systems that maximize both sensitivity and durability. These hybrid formats reduce degradation and enhance performance in real-world settings [50].

#### 2.2.4. AI-Assisted Sensing

Machine learning is increasingly being employed to improve biosensor accuracy. AI helps interpret complex datasets, minimizes false positives, and enables real-time molecular fingerprinting and automated decision-making [31]. These innovations are paving the way for next-generation biosensors that are highly adaptable, stable, and ready for field deployment across a range of industries.

### 2.3. Challenges in Stability, Specificity, and Scalability

Despite significant advancements, several limitations still hinder the widespread application of biorecognition elements in bionic sensors.

#### 2.3.1. Stability

Biological bioreceptors like enzymes and antibodies are susceptible to denaturation and degradation, affecting sensor shelf life and reliability. To improve stability, approaches such as immobilization, encapsulation, and the use of synthetic alternatives are under investigation [22,35,51].

#### 2.3.2. Specificity

High specificity is essential for biosensors to accurately detect only the target analytes and avoid cross-reactivity. Aptamers and molecularly imprinted polymers (MIPs) provide adjustable selectivity through sequence design and polymer imprinting, respectively [52,53].

#### 2.3.3. Scalability

Scaling up biosensor production remains challenging due to high costs, batch-to-batch variability, and regulatory constraints. Progress in automated manufacturing and standardized material synthesis is essential for commercial scalability [54].

## 3. Nanomaterials and Their Role in Enhancing Sensitivity and Selectivity in Biosensors

The integration of nanomaterials into biosensor technology has significantly enhanced their functional capabilities, enabling superior sensitivity, improved selectivity, effective signal amplification, and real-time detection performance [55]. Nanomaterials have unique characteristics such as a large surface area, distinctive electronic and optical properties, and the ability to be easily functionalized [56,57]. These properties make them ideal for developing compact and sensitive biosensors. Advances in materials like graphene, MXenes, carbon nanotubes, and quantum dots have broadened their use in diagnostics, environmental monitoring, and wearable devices. Recent research trends highlight how nanomaterials enhance signal transduction and detection performance, with hybrid systems enabling ultra-sensitive and multifunctional biosensors. These features are especially important for small molecule sensing, where signal amplification and surface interactions play a crucial role in achieving low detection limits.

### 3.1. Emerging Nanomaterials for Biosensing

Nanomaterials such as graphene, carbon nanotubes (CNTs), quantum dots (QDs), and MXenes are widely studied for biosensor development due to their adaptable chemical, optical, and electronic properties [58]. These materials enhance bioreceptor immobilization and promote efficient electron or photon transfer, resulting in stronger signals and lower detection limits [59,60].

#### 3.1.1. Graphene-Based Biosensors

Graphene is a single layer of carbon atoms arranged in a hexagonal pattern and is known for its excellent electrical conductivity, large surface area, and strong mechanical properties. These features make it useful in biosensors by enabling efficient electron transfer and providing a large surface for bioreceptor immobilization, improving signal detection and target recognition [61,62]. Graphene-based field-effect transistors (GFETs) have shown strong potential in detecting minute quantities of biomarkers. However, challenges such as difficult functionalization, high production costs, and scalability limit their broader use [28]. To address these issues, researchers are developing graphene composites, including graphene–MXene hybrids, to improve stability and surface modification. Despite their exceptional electrical and physicochemical properties, graphene-based biosensors still face challenges related to large-scale fabrication, surface functionalization reproducibility, and long-term operational stability in complex sensing environments.

#### 3.1.2. Carbon Nanotubes (CNTs) in Signal Transduction

Carbon nanotubes are cylindrical nanostructures derived from rolled graphene sheets and are categorized as single-walled (SWCNTs) or multi-walled (MWCNTs). Their superior electrical conductivity and high surface-to-volume ratio make them ideal candidates for sensitive biosensor development [63]. CNTs can be functionalized with biomolecules such as enzymes, antibodies, or fluorescent tags to improve selectivity and enable real-time detection [64]. For example, polyethylene glycol (PEG)-modified CNTs combined with Rituxan antibodies have been used to selectively detect CD20 receptors on B cells, facilitating high-resolution fluorescence imaging in cancer diagnostics [65]. Although CNT-based biosensors demonstrate high analytical sensitivity, issues related to nanotube aggregation, batch variability, and biocompatibility continue to limit their widespread commercial and clinical implementation.

#### 3.1.3. Quantum Dots (QDs) for Optical Biosensing

Quantum dots are semiconductor nanocrystals that exhibit bright, stable fluorescence and tunable emission properties based on particle size. These characteristics make them ideal for multiplexed optical biosensing and biomedical imaging [66]. QDs can be conjugated with aptamers, antibodies, or enzymes to improve molecular recognition and specificity [67]. In chemiluminescence resonance energy transfer (CRET) systems, QDs function as energy acceptors, enabling label-free detection with improved signal-to-noise ratios and reduced background interference [68,69].

#### 3.1.4. MXenes as Emerging 2D Nanomaterials

MXenes are a class of 2D transition metal carbides, nitrides, and carbonitrides that exhibit high electrical conductivity, chemical versatility, and biocompatibility [70]. Their surfaces can be tailored with functional groups such as hydroxyl, fluorine, or oxygen, offering tunable properties for various sensing applications [71]. MXene-based biosensors are being developed for wearable applications such as flexible electrochemical patches and sweat-based glucose monitoring systems. Their mechanical adaptability and efficient charge transport capabilities make them promising candidates for next-generation biosensor platforms [57]. Table 3 summarizes key properties of these emerging nanomaterials in biosensors.

#### 3.1.5. Electrochemical Signal Transduction Mechanisms in Small Molecule Biosensing

Electrochemical biosensors are among the most widely utilized platforms for small molecule detection due to their high sensitivity, rapid response, low cost, and compatibility with miniaturized and wearable systems. These biosensors operate by converting biochemical interactions occurring at the sensor interface into measurable electrical signals through electrochemical transduction mechanisms such as current, voltage, impedance, or conductance changes.

In most electrochemical biosensors, target recognition events trigger oxidation–reduction (redox) reactions at the electrode surface, generating electron transfer processes that can be quantitatively measured. Small molecules such as glucose, dopamine, lactate, toxins, and pharmaceutical compounds are particularly well suited for electrochemical analysis because many possess electroactive functional groups or can participate in enzymatically mediated redox reactions [29]. The efficiency of electron transfer between the analyte, bioreceptor, and electrode surface strongly influences sensor sensitivity and detection limits. Direct and mediated electron transfer mechanisms remain central to electrochemical biosensor performance, particularly in enzymatic sensing systems where efficient bioelectrocatalytic communication between enzymes and electrode surfaces is required for highly sensitive analyte detection [72].

Several electrochemical techniques are commonly employed in small molecule biosensing, including cyclic voltammetry (CV), differential pulse voltammetry (DPV), square wave voltammetry (SWV), and electrochemical impedance spectroscopy (EIS). CV is frequently used to investigate redox behavior and electron transfer kinetics, whereas DPV and SWV provide enhanced sensitivity for trace-level analyte detection by minimizing capacitive background currents. EIS is particularly valuable for monitoring interfacial charge transfer resistance and binding-induced impedance changes at modified electrode surfaces.

Nanomaterials such as graphene, CNTs, MXenes, metallic nanoparticles, and conductive polymers significantly enhance electrochemical sensing performance by improving electrode conductivity, increasing active surface area, and facilitating rapid electron transfer [28,29]. In addition, redox mediators and conductive polymer matrices can improve electron shuttling between immobilized bioreceptors and electrode surfaces, thereby amplifying electrochemical responses and improving signal stability. Conductive redox polymer systems have been extensively investigated for improving mediated electron transfer between enzymes and electrode interfaces, enabling enhanced electrochemical sensitivity, signal stability, and biosensing efficiency in electrochemical sensing platforms [73].

These strategies are especially important for small molecule detection in complex matrices, where weak analytical signals and nonspecific interference often limit conventional sensing performance. Recent advances integrating electrochemical biosensors with artificial intelligence further enable automated signal interpretation, adaptive calibration, and real-time molecular fingerprinting. Together, these developments are driving the emergence of highly sensitive, miniaturized, and intelligent electrochemical platforms for next-generation small molecule biosensing applications.

### 3.2. Hybrid Nanomaterials for Ultra-Sensitive Detection

The development of hybrid nanomaterials has opened new pathways for designing biosensors that leverage synergistic properties. By combining two or more nanomaterials, these hybrids achieve enhanced stability, sensitivity, and multi-functionality compared to individual components.

#### 3.2.1. Carbon Nanotube–MXene Composites

**Figure 6:**
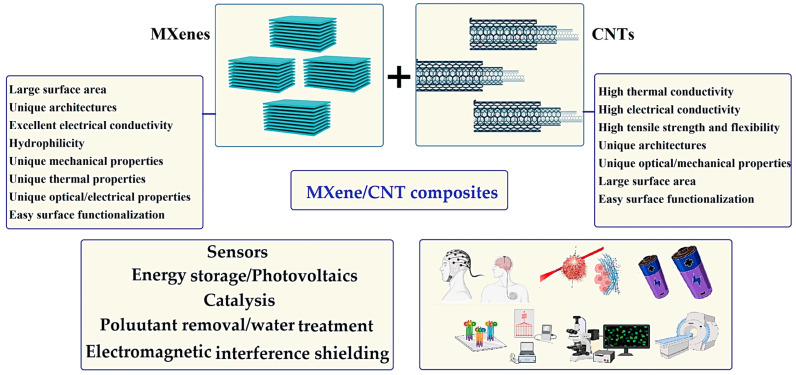
Schematic illustration of MXene/carbon nanotube (CNT) composite materials highlighting their structural features, physicochemical properties, and applications in sensing, energy storage, catalysis, water treatment, and biomedical technologies. Reproduced from [76], Nanomaterials, used under the terms of the Creative Commons Attribution (CC BY) license.

Hybrid composites of multi-walled carbon nanotubes and amino-functionalized Ti_3_C_2_ MXene have been used to construct sandwich-format biosensors for carcinoembryonic antigen (CEA) detection, a biomarker for colorectal cancer. These systems demonstrate broader detection ranges and lower detection limits than sensors based solely on MXenes [74]. The integration of CNTs and MXenes improves electrical conductivity and allows denser immobilization of biorecognition elements, thereby enhancing signal resolution and signal-to-noise performance. Beyond biosensing applications, MXene/CNT composites have also attracted attention in catalysis, energy storage, environmental remediation, and biomedical technologies because of their unique physicochemical properties. Recent advances in bioelectrode engineering, including nanostructured conductive interfaces and hybrid bioelectrocatalytic architectures, continue to improve electrochemical biosensor sensitivity, stability, and operational efficiency for complex analytical applications [75]. The structural features and multifunctional applications of MXene/CNT composites in sensing platforms are illustrated in Figure 6, adapted from Mohajer et al. [76].

#### 3.2.2. MXene Quantum Dots (MQDs)

**Figure 7:**
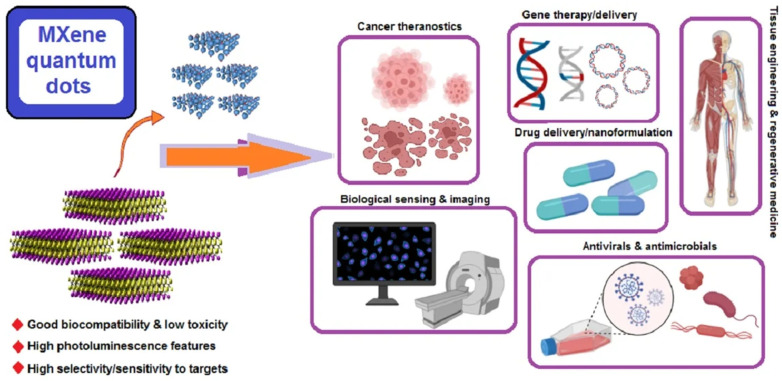
Overview of MXene quantum dots (MQDs), highlighting their physicochemical properties and emerging biomedical applications. The figure illustrates key MQD features, including nanoscale size, tunable surface chemistry, optical/electronic properties, and biocompatibility, as well as applications in biological sensing and imaging, cancer theranostics, gene therapy, drug delivery, and antimicrobial systems. Reproduced from [78], Nanomaterials, under the terms of the Creative Commons Attribution (CC BY) license.

MXene quantum dots (MQDs) are hybrid nanostructures that combine the electronic versatility of MXenes with the optical sensitivity of quantum dots. These materials exhibit enhanced fluorescence properties, tunable electrical conductivity, and excellent mechanical stability. MQDs are being explored for diverse biosensing applications, including tumor biomarker detection, heavy metal sensing, and compact point-of-care diagnostic systems [77]. The structural characteristics and emerging biomedical applications of MQDs are illustrated in Figure 7, adapted from [78].

Nanomaterials continue to play a transformative role in advancing biosensor performance by enhancing sensitivity, miniaturization, and functional integration. Ongoing research focused on scalability, biocompatibility, and multi-modal designs will be key to driving future innovations in biosensor technology. A comparative overview of the major nanomaterial-enabled biosensing platforms used for small molecule detection, including their key advantages, limitations, representative targets, and practical applications, is summarized in Table 4.

## 4. AI and Machine Learning in Bionic Sensor Data Processing

**Figure 8:**
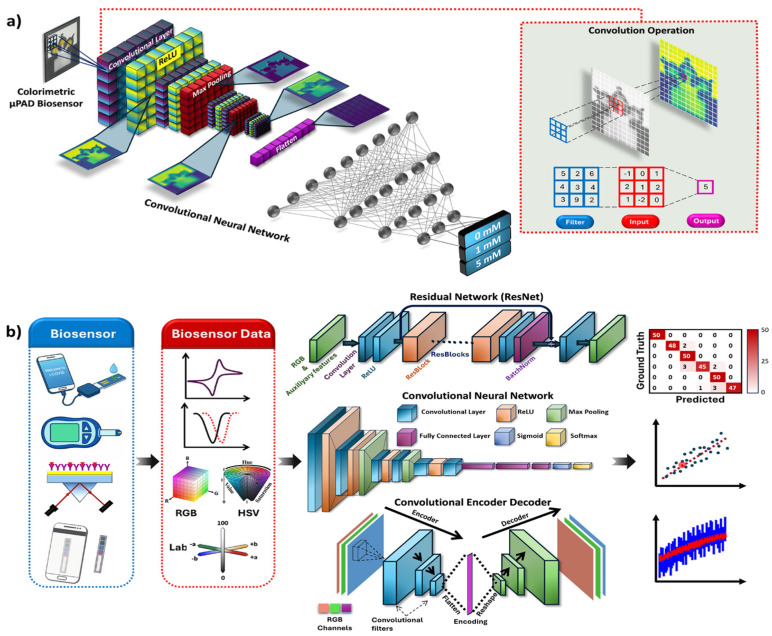
Overview of artificial intelligence-assisted biosensor data processing workflows. (**a**) Convolutional neural network (CNN) architectures applied to colorimetric and spectroscopic biosensor signals for feature extraction, denoising, and classification. (**b**) Integration of biosensor outputs with deep learning models, including CNN, ResNet, and encoder–decoder architectures, for molecular fingerprint recognition, adaptive calibration, and predictive analysis in complex sensing environments. Adapted from [84], Advanced Materials, under the terms of the Creative Commons Attribution (CC BY) license.

Artificial intelligence (AI) and machine learning (ML) are transforming biosensor data processing by improving sensor calibration, correcting measurement errors, enabling real-time pattern recognition, and supporting molecular fingerprinting [79,80,81]. These capabilities substantially enhance the accuracy, efficiency, and practical applicability of biosensing systems. In addition, AI-enhanced sensor networks are increasingly being applied in advanced diagnostics, environmental monitoring, and industrial systems to improve analytical reliability while reducing the need for manual data interpretation [82,83]. Typical AI-enabled biosensing workflows, including convolutional neural networks and deep learning architectures used for biosensor data analysis, are illustrated in Figure 8, adapted from [84].

The core contributions of artificial intelligence in biosensing can be categorized into three main areas: (1) adaptive calibration and error correction, (2) deep learning for molecular identification, and (3) integrated AI–biosensor systems for diagnostics and real-time monitoring. In the context of small molecule detection, AI plays a particularly important role in resolving weak signals, overlapping spectral features, and matrix-induced interference that are often challenging for conventional analytical methods. Table 5 summarizes deep learning applications in molecular biosensing.

### 4.1. Adaptive Calibration, Error Correction, and Pattern Recognition

Biosensors used in real-world settings often face challenges such as signal drift, noise interference, and hardware variability. AI-driven models play a key role in addressing these issues through real-time correction and adaptive calibration [90,91]. Adaptive learning algorithms, reinforcement learning (RL), and digital twin models are increasingly used to continuously improve the accuracy and stability of sensor systems [92,93,94]. For example, rolling-horizon calibration methods based on genetic algorithms allow sensors to adapt dynamically to changing environmental conditions, thereby enhancing precision in complex systems [95]. Reinforcement learning techniques, including Proximal Policy Optimization (PPO), have demonstrated effectiveness in improving sensor performance and optimizing energy efficiency [96,97]. Digital twins, which are virtual replicas of physical sensors, support predictive maintenance and self-learning correction by simulating sensor behavior under changing conditions [98]. These models are especially valuable in industrial and biomedical systems where real-time adaptability is essential.

Artificial intelligence also plays a key role in error compensation. Gaussian Process Regression (GPR) models can dynamically adjust sensor parameters in response to incoming data, reducing measurement deviations and improving reliability in precision applications such as metrology and laboratory diagnostics [99]. Web-based calibration frameworks such as GEMIMEG now incorporate artificial intelligence to streamline the creation of digital calibration certificates, supporting standard compliance in IoT-based sensor systems [100]. In addition, pattern recognition algorithms such as convolutional neural networks (CNNs) and object detection models like YOLOv4 are increasingly applied in robotic biosensors and portable devices. These systems automatically classify complex inputs, identify subtle patterns within sensor data, and effectively differentiate between signal and noise, thereby enhancing detection accuracy and reliability [101,102]. AI technologies are increasingly used to strengthen the cybersecurity of biosensors, enabling the detection of malicious threats and safeguarding critical health and environmental information [103,104,105].

### 4.2. Deep Learning for Real-Time Molecular Fingerprinting

Deep learning helps biosensors detect and classify molecular patterns in complex spectral and biochemical data, often without the need for extensive preprocessing [106,107]. These computational approaches are particularly valuable for small molecule sensing, where weak signals, overlapping spectral fingerprints, and matrix-induced interference often limit the performance of traditional analytical methods.

Deep neural networks (DNNs) combined with wavelet transforms help reduce Gaussian noise and correct baseline drift, enabling more accurate classification of weak signals [85]. Convolutional neural networks (CNNs), when applied to Raman spectroscopy, can process biological samples in real time without requiring prior feature extraction, thereby improving diagnostic speed and accuracy [86,87,108]. In biomedical applications, advanced models like ResNet-based CNNs have been used to identify cancer markers and bacterial extracellular vesicles (EVs), reliably distinguishing between healthy and diseased samples [88,89,109]. AI-powered electrochemical aptasensors have also shown enhanced precision in detecting neurodegenerative diseases and viral infections through highly specific molecular recognition [31,110]. Moreover, mobile biosensing platforms increasingly incorporate AI for point-of-care diagnostics. One-dimensional CNNs embedded in handheld Raman spectrometers outperform traditional matching methods by automating real-time molecular identification [30]. Artificial intelligence is particularly valuable in small molecule biosensing because many target analytes generate weak, overlapping, or noise-sensitive analytical signals within complex biological and environmental matrices. Deep learning models such as convolutional neural networks (CNNs) and recurrent neural networks (RNNs) can identify subtle electrochemical, optical, and spectroscopic signal patterns that are often difficult to distinguish using conventional analytical methods. For example, AI-assisted Raman and electrochemical biosensing systems have demonstrated improved detection accuracy for low-abundance metabolites, toxins, and pharmaceutical compounds by enabling signal denoising, spectral deconvolution, adaptive calibration, and automated molecular fingerprint recognition [30,31]. These approaches significantly enhance sensitivity and reduce false-positive responses in complex sensing environments where matrix interference and signal overlap remain major limitations.

### 4.3. AI-Powered Sensor Networks for Diagnostics and Monitoring

The integration of artificial intelligence (AI) with wireless sensor networks (WSNs) has facilitated the creation of intelligent biosensor ecosystems capable of distributed, real-time data analysis [111,112]. These systems are proving essential across sectors such as healthcare, agriculture, and environmental monitoring. In medical diagnostics, machine learning algorithms such as support vector machines (SVMs) and random forest classifiers (RFCs) have demonstrated high accuracy in detecting infectious diseases [113]. Additionally, emerging frameworks in explainable AI (XAI) are enabling clinicians and researchers to interpret model predictions, thereby increasing transparency in automated decision-making [114].

Point-of-care diagnostics now benefit from AI-enabled biosensors that autonomously assess physiological and biochemical markers, making early disease detection more accessible, even in remote or resource-limited settings [115,116]. In manufacturing, long short-term memory (LSTM) autoencoders have been employed for predictive fault diagnosis in sensor-driven production systems [117]. In agriculture, AI-enhanced biosensors support vertical farming by enabling climate control, pest prediction, and maintenance scheduling [118]. In industrial automation, the integration of AI with spectroscopy platforms such as surface-enhanced Raman spectroscopy (SERS) and localized surface plasmon resonance (LSPR) allows for pollutant detection at trace levels [119]. Furthermore, in environmental monitoring, AI-integrated biosensors enable real-time analysis of parameters like pH, oxidation–reduction potential, and turbidity, significantly improving wastewater management and water quality surveillance [25,120].

Overall, AI and machine learning are essential to advancing bionic sensors, enabling more accurate, scalable, and autonomous biosensing through adaptive calibration, deep learning, and real-time decision-making. Emerging innovations in edge AI, data fusion, and cybersecurity are set to drive the next generation of smart, energy-efficient biosensors across medical, industrial, and environmental fields. Despite substantial progress in AI-assisted biosensing, challenges related to data standardization, algorithm interpretability, model overfitting, and cybersecurity remain important barriers to reliable large-scale deployment.

## 5. Wearable and Implantable Bionic Sensors for Real-Time Monitoring

**Figure 9:**
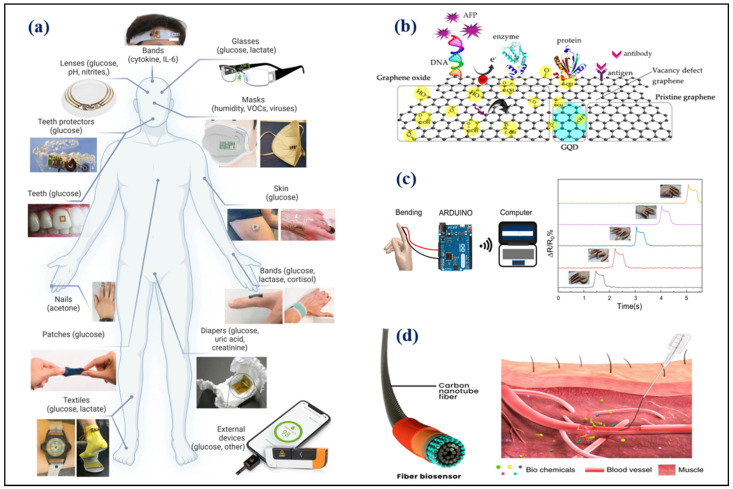
Representative wearable and implantable biosensor technologies for real-time health monitoring. (**a**) Wearable biosensors are integrated into contact lenses, glasses, masks, skin patches, textiles, and portable devices for monitoring physiological biomarkers. Adapted from [31], Biosensors, under CC BY license. (**b**) Graphene-based biosensing platforms for detection of biomolecules including DNA, proteins, enzymes, and antibodies. Adapted from [123], Sensors, under CC BY license. (**c**) Flexible biosensor systems are integrated with microcontrollers for signal acquisition and analysis. Adapted from [124] under CC BY 4.0 license. (**d**) Implantable fiber biosensors for in vivo biochemical monitoring. Adapted from [125], Applied Sciences, under CC BY license.

The rise in chronic diseases and the need for more personalized healthcare have led to fast progress in wearable and implantable biosensors [121]. These devices can track body signals and chemical markers continuously, giving real-time information that helps with earlier diagnosis, better treatment, and prevention [31]. Advances in self-powered biosensors further demonstrate the growing potential of autonomous electrochemical sensing systems for wearable and continuous monitoring applications [122]. As medicine becomes more focused on the individual, these biosensors are becoming important tools for supporting personalized care. Representative wearable and implantable biosensing platforms and their applications in health monitoring are illustrated in Figure 9.

These platforms are particularly well suited for continuous monitoring of clinically relevant biomarkers, including small molecules such as glucose, lactate, metabolites, and therapeutic compounds in biofluids. Advances in wearable and implantable biosensor engineering are enabling real-time biochemical analysis that supports personalized healthcare and precision medicine applications. Beyond conventional biomacromolecule monitoring, these biosensors are increasingly being designed for continuous small-molecule sensing, enabling dynamic detection of metabolites, therapeutic drugs, toxins, and environmental contaminants in complex physiological environments.

### 5.1. Wearable Biosensors for Continuous Health Monitoring

Wearable biosensors are designed to be worn on the body or integrated into clothing and accessories, enabling non-invasive, real-time monitoring of biomarkers from body fluids such as sweat, saliva, or tears [126]. They typically consist of a bioreceptor such as an enzyme, peptide, nucleic acid, or antibody that binds to a target analyte, and a transducer electrochemical or optical that converts this interaction into an electrical signal [16]. A leading application is glucose monitoring. In enzyme-modified field-effect transistors (EnFETs), the enzymatic reaction occurring at the sensing interface directly modulates the electrical characteristics of the semiconductor channel. For example, glucose oxidase (GOx) catalyzes the oxidation of glucose to gluconic acid, producing hydrogen peroxide (H_2_O_2_) and protons (H^+^). The generated charges alter the local electric field near the gate surface, resulting in redistribution of charge carriers within the semiconductor and measurable changes in drain–source current. Thus, analyte concentration can be quantified through current modulation without the need for labeling [26]. This mechanism underpins many emerging wearable glucose monitoring platforms designed for continuous and minimally invasive detection. With 14% of adults over 18 living with diabetes in 2022 [127], the demand for continuous glucose monitoring systems (CGMs) is rising. Traditional methods rely on intermittent blood sampling, lacking real-time data. As of May 2026, the U.S. Food and Drug Administration (FDA) has not authorized, cleared, or approved any smartwatches or smart rings intended to measure or estimate blood glucose levels independently. The FDA has issued a safety communication advising consumers against using such devices for blood glucose measurement due to concerns about their accuracy and reliability [127,128].

While FDA-approved CGMs offer continuous tracking, access remains unequal. Studies show that individuals from lower socioeconomic backgrounds face barriers to adoption, emphasizing the need for affordable, widely available wearable biosensor technologies [129]. Long-term signal stability, motion-induced artifacts, sweat interference, and power management remain important engineering challenges for wearable biosensors operating under dynamic physiological conditions.

### 5.2. Graphene-Based Nanomaterials in Wearable Biosensors

To improve comfort, wearability, and miniaturization, researchers are exploring nanomaterials, especially two-dimensional (2D) materials like graphene, for biosensor development. “Graphene”, offers exceptional electrical conductivity, mechanical strength, and surface area, making it ideal for wearable sensors [130,131]. Its strength comes from covalent carbon–carbon bonds, while its conductivity supports efficient signal transmission in biosensing. Biocompatibility and chemical stability further enhance their use in health monitoring systems [132].

### 5.3. Fabrication Methods for Graphene-Based Wearable Sensors

Multiple fabrication methods have been explored to produce graphene for wearable biosensors, each affecting the material’s quality, conductivity, and scalability. Chemical vapor deposition (CVD) is widely used, involving the decomposition of carbon-rich gases like methane to deposit graphene on a substrate. It is cost-effective and scalable but depends heavily on substrate type and produces chemical waste [133,134]. Mechanical or liquid-phase exfoliation separates graphene layers from graphite using adhesive tape or sonication. While low-cost and scalable, it often yields inconsistent layer thickness and limited quality control [135,136]. Laser-induced graphene (LIG) uses lasers to convert carbon-rich materials into porous graphene, offering an energy-efficient and eco-friendly approach, though defect density may affect long-term stability [137,138,139]. A group of researchers successfully developed a non-enzymatic wearable glucose biosensor using LIG with gold and nickel plating, enabling non-invasive, real-time glucose detection from sweat [140]. Table 6 compares different fabrication methods for graphene-based wearable biosensors.

### 5.4. Implantable Biosensors and Precision Medicine

While wearable biosensors offer non-invasive monitoring, implantable biosensors provide continuous, long-term tracking of critical biomarkers such as glucose, lactate, cardiac markers, and neurological signals [141]. These sensors, placed subcutaneously or within tissues and organs, are central to precision medicine by enabling personalized therapy and real-time drug response assessment [20,142]. However, key challenges include biocompatibility, power supply, and wireless communication. Long-term implantation can trigger immune responses or fibrosis, affecting sensor performance [143,144,145,146]. To address this, researchers are developing bioinert and bioresorbable materials to reduce immune reactions and extend device lifespan [147,148,149]. Power remains a limitation, as most systems rely on batteries, prompting interest in energy harvesting from body heat, movement, or biochemical reactions. For data transmission, technologies like Bluetooth Low Energy (BLE), near-field communication (NFC), and radio-frequency identification (RFID) are being optimized for seamless wireless communication without invasive retrieval [150].

### 5.5. Stimuli-Responsive Micro/Nanorobotic Biosensors

Emerging micro- and nanorobotic biosensors represent a new generation of movable sensing platforms capable of actively navigating complex biological and environmental environments for targeted detection applications. Unlike conventional static biosensors, these systems can autonomously or remotely move toward specific locations, enabling localized sensing in confined, dynamic, or difficult-to-access regions. Their active mobility significantly improves analyte accessibility, spatial resolution, and real-time monitoring capabilities, particularly in biomedical applications.

Micro/nanorobotic biosensors are commonly fabricated using stimuli-responsive materials that respond to external triggers such as magnetic fields, light, ultrasound, chemical gradients, or electric fields [151]. Among these approaches, magnetically actuated microrobots have gained substantial attention due to their precise remote controllability, biocompatibility, and potential for minimally invasive sensing and therapeutic applications. Recent advances have demonstrated magnetic microrobotic systems capable of multimodal airway sensing, adaptive navigation in dynamic environments, and targeted biomarker detection with improved sensitivity and operational flexibility [152,153].

In addition to biomedical diagnostics, micro/nanorobotic biosensors are being explored for environmental monitoring, localized pollutant detection, and precision drug delivery. Their integration with nanomaterials, electrochemical transducers, and artificial intelligence-assisted navigation systems may further enhance autonomous sensing, signal processing, and decision-making capabilities. Although challenges related to fabrication complexity, long-term biocompatibility, energy supply, and regulatory approval remain, stimuli-responsive microrobotic biosensors represent a promising future direction for next-generation small molecule sensing technologies.

Wearable and implantable biosensors are transforming healthcare through real-time, continuous monitoring of physiological and biochemical signals. Advances in nanomaterials like graphene are driving miniaturization and enhanced functionality, while new fabrication techniques are improving scalability and cost-effectiveness. Despite challenges such as biocompatibility and energy limitations in implantables, ongoing innovation is making these systems more reliable and accessible, supporting the future of personalized medicine.

## 6. Bionic Sensors in Clinical Diagnostics and Personalized Medicine

The word “Bionic” refers to the systems or artificial body parts that simulate or enhance natural biological functions, often by combining biology with electronics [154]. Bionic sensors are transforming clinical diagnostics and personalized healthcare by enabling fast, highly sensitive, and real-time monitoring of disease-related biomarkers [155]. These innovative biosensor technologies are revolutionizing healthcare by supporting early detection of diseases, continuous monitoring of patients, and the development of personalized treatment plans [156]. The combination of bionic sensors with nanotechnology, artificial intelligence (AI), and biocompatible materials has emerged as a vital advancement in clinical settings, with applications in cancer screening, infectious disease diagnosis, metabolic disorder monitoring, and precision medicine [22,31]. The following discusses the impact of bionic sensors in reshaping diagnostic practices, enhancing point-of-care testing (POCT), and contributing to progress in precision medicine and biomarker identification. While much of clinical biosensing focuses on macromolecular biomarkers, the detection of small molecule metabolites remains essential for real-time physiological monitoring and disease management.

### 6.1. Revolutionizing Disease Diagnosis: Cancer, Infectious Diseases, and Metabolic Disorders

#### 6.1.1. Cancer Diagnostics and Early Detection

Early-stage cancer diagnosis is strongly associated with improved therapeutic efficacy and higher patient survival rates [157]. Conventional diagnostic methods, including histopathological analysis and imaging techniques, frequently involve invasive interventions and extended processing durations [158]. Bionic sensors provide a non-invasive and highly sensitive approach for identifying circulating tumor DNA (ctDNA), exosomes, and protein biomarkers in bodily fluids such as blood, saliva, and urine [159]. Table 7 highlights how these innovations establish bionic sensors as a transformative technology in oncology, facilitating rapid, accurate, and personalized cancer diagnostics.

Electrochemical biosensors enable the highly sensitive and real-time detection of cancer-associated biomarkers, including carcinoembryonic antigen (CEA), alpha-fetoprotein (AFP), and prostate-specific antigen (PSA) [160,161]. Optical biosensors incorporating nanomaterials such as gold nanoparticles, quantum dots, and graphene-based substrates, facilitate highly sensitive cancer detection through fluorescence and surface-enhanced Raman spectroscopy (SERS) methodologies [162,163]. Liquid biopsy-based biosensors enable the identification of circulating tumor cells (CTCs) and exosomal RNA, offering a minimally invasive strategy for cancer diagnosis and prognostic evaluation [164].

#### 6.1.2. Infectious Disease Detection and Outbreak Monitoring

The worldwide impact of infectious diseases such as COVID-19, tuberculosis, and HIV has highlighted the critical demand for diagnostic tools that are rapid, portable, and economically accessible [165,166]. Bionic sensors enable real-time identification of pathogens, thereby enhancing disease monitoring and supporting timely responses to outbreaks [31]. Table 8 summarizes key advancements in bionic biosensor technologies for infectious disease diagnostics, highlighting emerging platforms that enable rapid, sensitive, and decentralized detection of viral and bacterial pathogens.

CRISPR-Cas-based biosensors offer highly specific recognition of nucleic acid sequences, enabling rapid and ultrasensitive detection of viral and bacterial pathogens at the single-molecule level [167,168]. Microfluidic lab-on-a-chip biosensors combine nucleic acid amplification with immunoassay methodologies, enabling swift and decentralized detection of viral RNA and bacterial DNA at the point of care [169,170]. Wearable biosensors monitor host immune activity and cytokine concentrations, offering continuous, real-time assessment of disease progression in critically ill individuals [17].

#### 6.1.3. Metabolic Disorder Management: Diabetes and Cardiovascular Diseases

Bionic sensor technologies are revolutionizing the monitoring and clinical management of chronic metabolic conditions, including diabetes, obesity, and cardiovascular disorders. Advances in bionic biosensors have enabled continuous and non-invasive monitoring of critical metabolic biomarkers. Continuous monitoring of indicators such as glucose, lactate, cholesterol, and ketones allows for real-time health tracking and timely interventions [17,171]. Table 9 summarizes representative sensor platforms that support real-time disease management and promote early intervention in conditions such as diabetes, obesity, and cardiovascular disease.

Continuous glucose monitoring (CGM) biosensors employ enzyme-coated electrodes to measure glucose concentrations in interstitial fluid, providing a non-invasive and real-time approach to diabetes management [154,172]. Wearable electrochemical biosensors monitor lactate and cholesterol concentrations, facilitating early identification of metabolic abnormalities and supporting tailored lifestyle interventions [171,173]. Smart tattoos and sweat-based biosensors utilize ion-selective electrodes to detect physiological biomarkers, providing a non-invasive alternative to conventional blood-based diagnostic methods [174,175]. Collectively, these bionic sensing platforms contribute to more effective disease management, improved patient adherence, and proactive lifestyle modifications in individuals with chronic metabolic disorders.

### 6.2. Point-of-Care Applications and Miniaturized Lab-on-Chip Devices

Point-of-care (POC) diagnostic technologies are designed to deliver laboratory-grade analytical performance directly at the patient site, reducing dependence on centralized facilities and minimizing sample handling and processing time [170]. (Miniaturized lab-on-a-chip (LOC) systems combine microfluidic devices, biosensing components, and automated data processing to enable fast and precise diagnostic testing at the point of care [176]. Table 10 summarizes lab-on-a-chip (LOC) technologies that enable real-time diagnostics, reduce turnaround times, and support decentralized healthcare.

**Figure 10:**
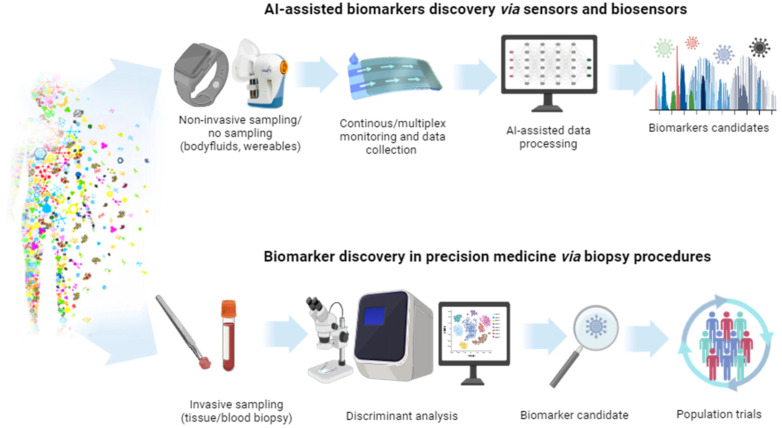
Comparison of biomarker discovery workflows in precision medicine. AI-assisted biosensing enables non-invasive sampling, continuous physiological monitoring, and automated data processing for identification of biomarker candidates, whereas traditional approaches rely on invasive biopsy sampling followed by laboratory analysis and clinical validation. Adapted from [31], Biosensors, under the terms of the Creative Commons Attribution (CC BY) license.

Microfluidic paper-based analytical devices (µPADs) offer a simple and cost-effective approach for diagnosing infectious, metabolic, and inflammatory diseases, using colorimetric or electrochemical signals to detect biomarkers [177,178]. Smartphone-integrated biosensors analyze easily accessible samples like saliva, sweat, and urine, enabling AI-driven remote diagnostics and continuous patient monitoring without the need for hospital visits [179]. Point-of-care immunosensors incorporating nanoparticles and quantum dots (QDs) enhance the sensitivity of lateral flow assays (rapid, paper-based diagnostic devices commonly used for detecting infections such as COVID-19 and HIV) by improving signal amplification and detection accuracy [170,180]. Beyond miniaturized diagnostic hardware, recent advances in AI-assisted biosensing are also transforming biomarker discovery and precision medicine workflows. The integration of biosensors with artificial intelligence is accelerating biomarker discovery by enabling non-invasive sampling, continuous physiological monitoring, and automated data analysis. Figure 10 illustrates the workflow of AI-assisted biomarker discovery using biosensors compared with traditional biopsy-based biomarker identification approaches used in precision medicine.

#### Role of Bionic Sensors in Precision Medicine and Biomarker Discovery

Bionic sensors are increasingly advancing toward precision medicine, an approach that tailors treatment strategies to individual patient profiles based on genetic, molecular, and physiological characteristics while enabling continuous and personalized real-time data collection [181]. The integration of biosensors with artificial intelligence (AI) and big data analytics is accelerating biomarker discovery and enabling more personalized and effective therapeutic interventions [182]. Table 11 summarizes recent advances in bionic sensor technologies that support identification of patient-specific disease signatures, facilitating targeted therapies, minimizing adverse effects, and improving treatment outcomes in areas such as oncology, neurology, and autoimmune disorders.

Multiplexed biosensors enable the simultaneous detection of multiple disease biomarkers, supporting detailed molecular profiling to guide personalized medical [17,183,184]. AI-integrated biosensors process real-time patient data to enable predictive modeling, facilitating early disease detection and individualized therapeutic response assessment [182]. Organoid-based biosensors replicate patient-specific tissue microenvironments, enabling functional drug screening and the optimization of personalized treatment strategies [185,186]. Bionic sensors are reshaping clinical diagnostics and personalized medicine by improving disease detection, enabling real-time monitoring, and supporting targeted treatments. Their integration with nanotechnology, AI, and microfluidics is driving the development of non-invasive, affordable, and patient-focused healthcare solutions. As research progresses, these sensors are set to play a key role in early diagnosis, treatment precision, and disease prevention, ultimately leading to more effective and personalized care.

## 7. Environmental and Food Safety Applications of Bionic Sensors

**Figure 11:**
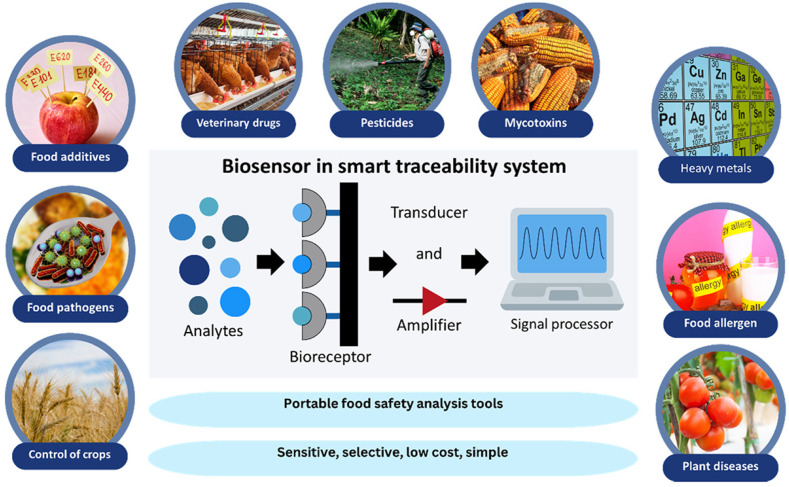
Biosensors in smart food traceability systems for monitoring contaminants and quality indicators across the food supply chain. Biosensors detect analytes such as food additives, veterinary drugs, pesticides, mycotoxins, heavy metals, pathogens, allergens, and plant disease markers through bioreceptor–transducer interactions, enabling rapid and portable food safety analysis. Adapted from [190], Bioengineered, under the terms of the Creative Commons Attribution (CC BY) license.

Bionic sensors are transforming environmental and food safety monitoring by enabling rapid, real-time, and highly sensitive detection of contaminants and quality indicators [187,188]. These systems convert biochemical, physical, or chemical signals into measurable outputs, allowing continuous assessment of food quality and environmental conditions [2,24]. Applications include detection of foodborne pathogens, toxins, pesticides, allergens, and veterinary drug residues, as well as smart packaging and portable field-deployable sensing systems that support public health, sustainability, and innovation in the food industry [189]. Figure 11 illustrates the role of biosensors in smart food traceability systems for monitoring contaminants such as pathogens, pesticides, veterinary drugs, heavy metals, allergens, and mycotoxins. Many of these applications rely heavily on the detection of small-molecule contaminants, including pesticides, toxins, additives, and volatile compounds, often within highly complex environmental and food matrices.

### 7.1. Detection of Toxins, Pesticides, and Pollutants in Food and Water

Bionic sensors are transforming food safety by enabling rapid detection of microbial contaminants, chemical toxins, and hazardous residues in food and water [191,192]. Traditional methods like chromatography and spectrophotometry, though accurate, require costly equipment, complex sample preparation, and trained personnel. In contrast, biosensors offer fast, cost-effective, and portable alternatives [193,194].

A major food safety concern is acrylamide, a carcinogen formed during high-temperature cooking [195]. Voltammetric biosensors using hemoglobin-modified carbon paste electrodes have detected acrylamide at concentrations as low as 1.2 × 10^−10^ M, showing high sensitivity in samples like fried potatoes [196].

In pesticide detection, optical fiber biosensors using acetylcholinesterase (AChE) and bromothymol blue (BTB) in sol-gel matrices have enabled real-time, label-free sensing of chlorpyrifos with minimal interference [197,198]. Gold nanoparticle-based biosensors also effectively detect biogenic amines, indicators of spoilage in meat and seafood [199]. Commercial systems like the Food Sentinel System, featuring barcode-based membrane sensors, identify pathogens such as *E. coli* and *Salmonella* directly on packaging [200]. Similarly, ToxinGuard^®^ packaging visually signals the presence of bacterial toxins, improving traceability and contamination prevention [201]. ToxinGuard™ uses immunoassay-based packaging with embedded antibodies that react with bacterial toxins, producing a visible color change on polyethylene film to indicate contamination [202]. Table 12 summarizes different applications of bionic sensors in food safety and packaging.

### 7.2. Smart Packaging and Real-Time Food Quality Monitoring

Integrating nanotechnology with biosensors in smart packaging has improved food safety through real-time detection of spoilage and contamination [203]. Nano-biosensors embedded in packaging materials can sense temperature, moisture, and pH, while also enhancing barriers and antimicrobial properties [204]. Colorimetric and electrochemical sensors detect freshness markers like acetic acid, n-butyrate, and biogenic amines [205]. For example, hydrogel-coated pH sensors monitor fish spoilage via total volatile basic nitrogen (TVB-N) levels [206]. Some smart materials respond to environmental changes such as humidity or gas composition by changing color or emitting electronic signals and may include tamper-evident or antimicrobial features [207,208]. However, regulatory gaps, biodegradability, and sensor safety remain key challenges [209].

### 7.3. Field-Deployable Biosensors for Environmental Hazard Detection

Field-Deployable or Portable bionic sensors are advancing environmental monitoring by enabling on-site detection of hazardous substances in air, water, and soil, especially in remote or resource-limited areas [210,211]. Graphene-based electrochemical biosensors show high sensitivity for heavy metals like lead (Pb), mercury (Hg), and cadmium (Cd) in drinking water, offering fast responses and ultra-low detection limits [29]. Enzyme-based air quality sensors detect VOCs, nitrogen oxides, and greenhouse gases, supporting real-time pollution tracking [212,213,214]. In agriculture and industry, biosensors monitor soil contaminants such as pesticides and heavy metals [215]. Microbial biosensors are also being adapted for in-field soil and waste monitoring [216]. Overall, bionic sensors are revolutionizing food and environmental safety by providing rapid, real-time, and specific detection of contaminants, from acrylamide in foods to pollutants in water and air. These technologies reduce reliance on laboratory testing while improving traceability and responsiveness. Future innovations will focus on enhancing sensitivity, scalability, and eco-compatibility, with AI-integrated sensor networks and biodegradable smart packaging shaping the next generation of sustainable solutions. Despite significant progress in environmental biosensing, achieving reliable long-term performance under highly variable field conditions remains challenging due to matrix complexity, sensor fouling, and environmental instability.

## 8. Challenges in Bionic Sensor Technologies: Scalability, Cost, and Ethical Considerations

Bionic sensors hold transformative potential across healthcare, environmental safety, and industry. However, widespread adoption is hindered by challenges in manufacturing scalability, cost, regulatory uncertainty, and ethical issues related to AI and implantable technologies. These challenges are particularly pronounced in small molecule sensing, where low signal intensity and high background interference complicate reliable detection.

### 8.1. Scalability and Standardization Barriers

Mass production faces hurdles due to material limitations, complex fabrication, and sensitivity to environmental conditions [217]. Materials like Shape Memory Alloys (SMAs) offer promise but suffer from fatigue and deformation, reducing long-term reliability [218,219]. Environmental instability and precision-dependent design also complicate large-scale deployment [220]. The absence of open-access Raman spectral databases limits generalizability in AI-based biosensors. Proprietary datasets restrict algorithm training, risking overfitting and poor performance across diverse settings [221]. Regulatory challenges also stem from the use of opaque AI models, such as deep learning and recurrent neural networks, which raise concerns around interpretability and safety, delaying clinical approval [222].

### 8.2. Benchmarking Challenges and Unmet Needs in Small Molecule Biosensing

Despite major technological progress, the field of small molecule biosensing still lacks standardized benchmarking frameworks for objectively evaluating sensor performance across different studies and application settings. Biosensor platforms are often tested using highly variable experimental conditions, analyte concentrations, matrices, and performance metrics, making direct comparison between sensing technologies difficult. Parameters such as limit of detection (LOD), sensitivity, selectivity, response time, stability, and reproducibility are frequently reported using inconsistent methodologies, limiting accurate assessment of real-world applicability and technological advancement.

Another major unmet need involves long-term operational stability and reproducibility in complex biological and environmental environments. Many biosensors demonstrate excellent analytical performance under controlled laboratory conditions but experience substantial reductions in sensitivity and reliability when exposed to real-world matrices containing proteins, salts, metabolites, lipids, or environmental contaminants. Matrix interference, biofouling, nonspecific adsorption, and signal drift therefore remain significant barriers to practical implementation.

Although artificial intelligence and machine learning algorithms have improved biosensor data analysis and signal interpretation, additional challenges remain regarding data quality, model interpretability, overfitting, and algorithmic reliability. Many AI-assisted biosensing systems require extensive high-quality datasets for robust training and validation, while insufficiently generalized models may produce unstable predictions in dynamic real-world conditions. In addition, concerns related to cybersecurity, wireless data transmission, and patient privacy continue to emerge as increasingly important considerations for cloud-connected biosensing platforms.

Future progress in the field will require greater emphasis on standardized validation protocols, scalable manufacturing strategies, long-term biocompatibility, and regulatory harmonization. Advances in self-powered biosensors, flexible bioelectronics, multimodal sensing systems, and AI-assisted adaptive calibration may further improve the practical deployment of next-generation biosensors for small molecule detection in healthcare, environmental monitoring, food safety, and personalized medicine applications.

### 8.3. Cost-Effectiveness and Resource Barriers

Economic constraints remain significant. AI-powered biosensors often depend on costly software, specialized hardware, and high-performance computing [31]. Raman spectrometers add further expense due to fabrication and intensive data processing needs [223,224]. In pharmaceutical development, AI can speed up biosensor-aided drug discovery, but high clinical trial costs and prolonged regulatory timelines often offset these benefits [156].

### 8.4. Ethical and Social Considerations

Ethical concerns revolve around data privacy, cybersecurity, and bias in AI algorithms [225]. Implantable biosensors gather sensitive data, posing risks of misuse and unauthorized access [27]. Many AI models work like a “black box,” meaning it’s hard to understand how they make decisions, which can reduce trust, especially in healthcare. If the training data isn’t diverse, the AI can become biased, leading to unequal treatment or wrong diagnoses. This highlights the importance of building fair and inclusive algorithms [226,227]. Social and cultural concerns also limit acceptance, as some people worry about altering the human body and losing personal privacy [228].

To advance bionic sensor technologies, multidisciplinary efforts must target technical, economic, and ethical barriers. Priorities include developing durable, low-cost materials, building open-access datasets, designing interpretable AI models, and creating robust regulatory frameworks. Addressing these challenges will support scalable, ethical, and equitable deployment across critical sectors.

## 9. Future Directions and Emerging Trends in Bionic Sensor Technologies

Bionic sensor technologies are undergoing rapid transformation, driven by advances in multi-target detection, secure data management, and energy-autonomous platforms. These innovations are expected to reshape healthcare, environmental surveillance, and biotechnology by enhancing diagnostic precision, patient autonomy, and data integrity. This section explores three major trends defining the future of bionic sensors: (1) next-generation hybrid detection platforms, (2) blockchain-enabled data systems, and (3) self-powered autonomous sensors. Future developments are expected to further enhance the capability of bionic sensors for multiplexed and high-throughput small molecule detection in real-world settings. Table 13 summarizes the emerging trends in bionic sensor technology.

### 9.1. Multi-Target Sensors and Hybrid Detection Systems

Traditional biosensors typically focus on detecting single analytes. However, next-generation platforms are being engineered to simultaneously track multiple biomarkers, increasing diagnostic power and efficiency [31,156]. These hybrid systems combine various sensing modalities such as optical, electrochemical, and mechanical, within a single unit to expand biosensor functionality.

#### 9.1.1. CRISPR/Cas-Based Multi-Analyte Detection

Recent developments in CRISPR/Cas biosensing enable precise DNA-based diagnostics [229,230]. Although current systems often require nucleic acid amplification, researchers are exploring multiplexed designs using multiple guide RNAs (gRNAs) to detect a range of antibiotic resistance genes in parallel [231]. These advancements open the door to quantitative, portable, and programmable biosensors.

#### 9.1.2. Nanomaterial-Enhanced Paper-Based Sensors

Paper-based biosensors are gaining attention for point-of-care diagnostics, especially in resource-limited settings. When integrated with nanomaterials, these systems demonstrate enhanced stability and multiplexing capabilities [232,233]. A recent prototype achieved simultaneous detection of various antibiotics in a single test, demonstrating the viability of low-cost, multi-analyte biosensing platforms [234].

#### 9.1.3. Hybrid Electronic Support Measures (ESM)

Multi-sensor ESM technologies show promise in medical and environmental diagnostics. By integrating radar and photoelectric sensors, hybrid ESM systems outperform single-sensor setups in accuracy and target tracking. These platforms offer real-time monitoring across physiological and environmental conditions, suggesting strong potential for next-gen wearable and remote sensing devices [235].

### 9.2. Blockchain Integration for Secure Data Management

As biosensors increasingly generate sensitive personal and environmental data, securing this information is critical. Blockchain offers a decentralized, transparent solution to ensure integrity, traceability, and access control for biosensing data [236].

#### 9.2.1. Decentralized Storage and Privacy

Blockchain enables peer-to-peer data exchange without reliance on centralized servers. In diagnostics, this ensures that patient data remain secure and verifiable while enabling real-time sharing among authorized users [237]. Blockchain integration allows biosensors to operate with enhanced security protocols for telemedicine and remote diagnostics [238,239].

#### 9.2.2. AI and Blockchain Synergy

Combining blockchain with AI analytics improves both data security and clinical decision-making. In chronic disease management, blockchain-secured biosensors allow encrypted health data to be analyzed by AI algorithms [240,241]. For example, in cardiovascular diagnostics, such systems clean, store, and analyze patient data via predictive modeling, offering real-time, privacy-preserved insights [242].

### 9.3. Self-Powered, Bioengineered, and Autonomous Sensors

Future biosensors aim to be fully autonomous, eliminating the need for external power sources and enabling long-term, real-time monitoring. Innovations in energy harvesting are central to this shift, with new devices powered by solar, biochemical, and mechanical energy [243,244,245].

#### 9.3.1. Perovskite Solar-Powered Wearables

A major breakthrough in wearable biosensing has been achieved with the integration of perovskite solar cells. These self-powered devices monitor multiple biomarkers, including glucose, pH, sweat rate, and temperature, continuously, without batteries. Their high photoconversion efficiency and miniaturization potential make them ideal for biomedical wearables [246,247].

#### 9.3.2. Dye-Sensitized Solar Cells (DSSCs)

DSSCs represent another emerging power source. In a recent application, researchers developed a DSSC-based biosensor capable of detecting sarcosine, a biomarker for prostate cancer. The system employed colorimetric shifts as visual indicators, requiring minimal power and functioning effectively in low-light environments [248].

#### 9.3.3. Enzymatic and Mechanical Energy Harvesting

Beyond solar technologies, biosensors are also being powered through enzymatic biofuel cells, triboelectric nanogenerators (TENGs), and piezoelectric materials. These mechanisms harvest energy from biochemical reactions or body motion, supporting wearable and implantable sensor applications [249,250]. Such developments are paving the way for biosensors that are not only portable and autonomous but also environmentally sustainable.

The next generation of bionic sensors will be defined by convergence, merging hybrid detection, AI-enhanced analytics, secure blockchain frameworks, and sustainable energy solutions. These innovations will expand the capabilities of biosensing technologies while addressing existing limitations in power, privacy, and scalability. Continued interdisciplinary research is essential to transition these technologies from lab prototypes to real-world applications, unlocking their full potential across healthcare, industry, and environmental sectors.

## 10. Conclusions

Bionic sensor technologies represent a powerful blend of biology, engineering, nanotechnology, and data science that is transforming how we detect and respond to biochemical signals in real time. This review has explored the key developments and future directions of these advanced sensing systems, with a focus on their mechanisms, recent innovations, and wide-ranging applications in healthcare, environmental monitoring, and food safety.

The integration of biological and synthetic recognition elements such as enzymes, antibodies, aptamers, and molecularly imprinted polymers has greatly improved the specificity and flexibility of biosensors. Advances in nanomaterials like graphene, carbon nanotubes, quantum dots, and MXenes have pushed the limits of sensitivity and miniaturization. These nanomaterial-enhanced biosensors now enable real-time, ultra-sensitive detection, extending their use from clinical settings to field-based environmental applications.

Artificial intelligence and machine learning are reshaping how biosensor data is processed, allowing for smarter calibration, pattern recognition, and molecular analysis. When combined with blockchain technologies, these systems also offer secure, transparent, and privacy-preserving data management. Together, these digital tools support the development of remote, predictive, and intelligent biosensing platforms.

Wearable and implantable biosensors have made continuous, non-invasive health tracking possible, providing real-time feedback for managing chronic conditions, improving workplace safety, and enabling personalized care. These devices are evolving from single-function tools into multifunctional systems that can monitor multiple biological signals at once. In parallel, food safety and environmental monitoring are being transformed by low-cost, paper-based, optical, and electrochemical biosensors that can quickly and accurately detect harmful substances like toxins, pathogens, and pollutants.

Despite this progress, challenges remain. Large-scale production, long-term biocompatibility, stability, and affordability continue to limit broader adoption. Ethical issues, particularly regarding implantable devices and AI-driven diagnostics, raise important questions about data privacy, fairness, and societal acceptance. Addressing these challenges will require close collaboration between researchers, clinicians, engineers, regulators, and ethicists. Notably, their impact is particularly significant in addressing the long-standing challenges associated with small molecule detection in complex, real-world environments.

Looking ahead, the future of bionic sensors lies in their continued integration with next-generation materials, decentralized data systems, and autonomous energy sources. Innovations in self-powered sensors using perovskite solar cells, biofuel cells, and triboelectric nanogenerators will improve device independence and sustainability. The rise of hybrid detection platforms, AI-blockchain synergy, and bioengineered smart interfaces will further extend the impact of bionic sensors in diagnostics, precision agriculture, and environmental protection.

In summary, bionic sensors are evolving into intelligent, adaptive systems that do more than just measure. They actively guide decision-making and connect users to real-time biological and environmental information. Their development marks a shift toward more proactive, personalized, and connected approaches to health, safety, and sustainability. With continued innovation, responsible oversight, and interdisciplinary collaboration, bionic sensor technologies will play a central role in building a healthier and smarter future.

## Figures and Tables

**Table 1:** Classification and Applications of Wearable, Placeable, and Implantable Biosensors.

Type	Definition	Common Applications	References
**Wearable**	Non-invasive sensors integrated into accessories, patches, or textiles	Health monitoring (heart rate, glucose, temperature), workplace safety, chronic disease management	[15,16,17]
**Placeable**	Portable or semi-fixed sensors used in external environments or on surfaces	Environmental monitoring (air/water quality), food quality control, pathogen detection	[14,18]
**Implantable**	Devices embedded inside the body for long-term use	Continuous glucose monitoring, cardiac biomarker tracking, brain signal analysis, precision medicine	[19,20,21]

**Table 2:** Comparative Features of Common Bioreceptors Used in Bionic Sensors (summarized from sources cited in Section 2.1).

Feature	Antibodies	Enzymes	Aptamers	MIPs
**Source**	Biological	Biological	Synthetic nucleic acids	Synthetic polymers
**Specificity**	High	High	High (customizable)	Moderate to High
**Stability**	Moderate	Low–Moderate	High	Very High
**Cost**	High	Moderate	Low–Moderate	Low
**Scalability**	Limited	Moderate	High	High
**Target Types**	Antigens	Substrates	Small molecules, proteins	Small molecules

**Table 3:** Key Properties of Emerging Nanomaterials in Biosensors.

Nanomaterial	Key Features	Biosensing Applications	Challenges	References
**Graphene**	High conductivity, large surface area	GFETs, biomarker detection	Cost, functionalization limitations	[28,61,62]
**CNTs**	High aspect ratio, conductivity	Fluorescent imaging, electrochemical sensing	Aggregation, reproducibility	[63,64,65]
**Quantum Dots**	Tunable emission, photostability	Optical biosensing, CRET systems	Cytotoxicity, surface passivation	[66,67,68,69]
**MXenes**	Biocompatible, modifiable surfaces	Wearable and flexible biosensors	Oxidation, synthesis complexity	[57,70,71]

**Table 4:** Comparative analysis of emerging nanomaterial-enabled biosensing platforms for small molecule detection.

Biosensing Platform	Key Advantages	Major Limitations	Small Molecule Targets	Typical Applications	References
**Graphene-based biosensors**	High electrical conductivity, large surface area, rapid electron transfer	High fabrication cost, difficult surface functionalization, scalability challenges	Glucose, dopamine, pesticides, toxins	Wearable sensing, electrochemical diagnostics	[28,62]
**Carbon nanotube (CNT)-based** **biosensors**	Excellent sensitivity, high aspect ratio, strong signal amplification	Aggregation issues, reproducibility limitations	Neurotransmitters, heavy metals, pharmaceutical compounds	Environmental and biomedical monitoring	[63,64]
**Quantum dot (QD)-based** **biosensors**	Tunable fluorescence, strong optical sensitivity, multiplexing capability	Potential cytotoxicity, surface instability	Drug molecules, metabolites, food contaminants	Optical biosensing and bioimaging	[67,68]
**MXene-based biosensors**	High conductivity, tunable surface chemistry, excellent electrochemical responsiveness	Oxidation susceptibility, synthesis complexity	Glucose, lactate, environmental pollutants	Flexible and wearable biosensors	[70,71]
**Hybrid nanocomposite biosensors**	Synergistic signal amplification, improved selectivity and stability	Complex fabrication processes	Small metabolites, toxins, multi-analyte systems	Point-of-care diagnostics and multifunctional sensing	[74,77]

**Table 5:** Deep Learning Applications in Molecular Biosensing.

Model Type	Application	Benefits	Reference
**DNN + Wavelet Transform**	Spectral denoising	Enhanced low-signal classification	[85]
**CNN**	Real-time Raman analysis	Faster, more accurate diagnostics	[86,87]
**ResNet-CNN**	Cancer and EV detection	Improved robustness, higher precision	[88,89]
**1D CNN**	Handheld molecular identification	Automated and portable diagnostics	[30]

**Table 6:** Comparison of Graphene Fabrication Methods for Wearable Biosensors.

Method	Advantages	Limitations	Suitability for Wearables
**Chemical Vapor Deposition**	Cost-effective, scalable	Substrate dependent, chemical waste	High, with optimized substrates
**Mechanical/Liquid Exfoliation**	Inexpensive, simple	Poor layer control	Moderate, with quality variation
**Laser-Induced Graphene**	Energy-efficient, eco-friendly	Defect density, long-term durability	High, for disposable or short-term use

**Table 7:** Representative Examples of Advanced Bionic Sensors for Cancer Diagnostics.

Type of Bionic Sensor	Target Analytes/Biomarkers	Advantages	Reference
**Electrochemical Biosensors**	Carcinoembryonic antigen (CEA), alpha-fetoprotein (AFP), prostate-specific antigen (PSA)	Exceptional sensitivity; real-time monitoring; label-free detection	[160,161]
**Nanomaterial-based Optical** **Biosensors**	Various cancer biomarkers (via gold nanoparticles, quantum dots, graphene platforms)	Ultra-sensitive detection; supports fluorescence and SERS-based techniques	[162,163]
**Liquid Biopsy** **Biosensors**	Circulating tumor cells (CTCs), exosomal RNA	Minimally invasive; enables early detection and prognosis monitoring	[164]

**Table 8:** Emerging Bionic Biosensor Technologies for Infectious Disease Diagnostics.

Biosensor Type	Functionality/Target	Key Advantages	Reference
**CRISPR-Cas Biosensors**	Detection of viral and bacterial nucleic acids	Ultra-specific recognition; single-molecule sensitivity; rapid identification	[167,168]
**Microfluidic Lab-on-Chip Devices**	Integrated nucleic acid amplification and immunoassays for viral RNA and bacterial DNA	Portable; rapid diagnostics; suitable for point-of-care applications	[169,170]
**Wearable** **Biosensors**	Monitoring host immune responses and cytokine levels	Continuous real-time tracking of disease progression, especially in critically ill patients	[17,171]

**Table 9:** Representative Bionic Sensor Technologies for Managing Chronic Metabolic Disorders.

Sensor Type	Target Biomarkers	Key Advantages	Reference
**Continuous Glucose Monitoring (CGM)**	Glucose (in interstitial fluid)	Enzyme-functionalized electrodes; real-time, non-invasive diabetes monitoring	[154,172]
**Wearable Electrochemical Biosensors**	Lactate, cholesterol	Early detection of metabolic dysfunctions; supports personalized lifestyle and therapeutic decisions	[171,173]
**Smart Tattoos & Sweat-Based Sensors**	Electrolytes, glucose, lactate (via ion-selective electrodes)	Non-invasive biomarker detection through sweat; alternative to conventional blood assays	[174,175]

**Table 10:** Emerging Lab-on-a-Chip and POC Biosensor Platforms for Decentralized Diagnostics.

Technology	Sample Type/Target Application	Key Features/Advantages	Reference
**Microfluidic Paper-based Analytical** **Devices (µPADs)**	Blood, saliva; infectious, metabolic, and inflammatory disease diagnostics	Low-cost, portable; uses colorimetric and electrochemical detection methods	[177,178]
**Smartphone-Integrated Biosensors**	Saliva, sweat, urine; remote diagnostics and monitoring	AI-assisted analysis; supports telemedicine and real-time patient monitoring	[179]
**POC Immunosensors with Nanoparticles/QDs**	Blood or serum; COVID-19, HIV, and other infectious diseases	Enhanced sensitivity of lateral flow assays through nanoparticle and quantum dot integration	[170,180]

**Table 11:** Advanced Bionic Sensor Technologies Supporting Precision Medicine.

Technology	Key Functionality	Clinical Application/Advantage	Reference
**Multiplexed Biosensors**	Simultaneous detection of multiple disease biomarkers	Enables comprehensive molecular profiling for individualized treatment planning	[17,183,184]
**AI-Powered Biosensors**	Real-time data analysis and predictive modeling	Supports early disease detection and tailored drug response predictions	[182]
**Organoid-Based** **Biosensors**	Mimic patient-specific tissue microenvironments for functional testing	Facilitates personalized drug screening and therapy optimization based on patient-derived cells	[185,186]

**Table 12:** Applications of Bionic Sensors in Food Safety and Packaging.

Application	Target Analyte	Sensor Type	Sample Matrix
**Acrylamide detection**	Acrylamide	Hemoglobin-modified voltammetric biosensor	Fried food products
**Pesticide monitoring**	Chlorpyrifos	AChE-BTB optical fiber biosensor	Water, produce
**Spoilage detection**	Biogenic amines, TVB-N	Gold nanoparticle, hydrogel pH sensors	Meat, seafood
**Pathogen sensing**	*E. coli*, *Salmonella*	Barcode-based membrane biosensors	Food packaging

**Table 13:** Summary of Emerging Trends in Bionic Sensor Technologies.

Trend/Technology	Subcategory	Key Features	Use Case/Impact
**Multi-Target Sensors and Hybrid Detection Systems**	CRISPR/Cas-Based Multi-Analyte Detection	Precise DNA diagnostics; multiplexed detection of antibiotic resistance genes using multiple gRNAs	Portable, programmable biosensors for rapid and specific pathogen detection
**Multi-Target Sensors and Hybrid Detection Systems**	Nanomaterial-Enhanced Paper-Based Sensors	Low-cost; enhanced stability; detects multiple antibiotics	Accessible diagnostics in resource-limited settings
**Multi-Target Sensors and Hybrid Detection Systems**	Hybrid Electronic Support Measures (ESM)	Combines radar and photoelectric sensors; high accuracy; real-time tracking	Wearable and remote sensing for medical and environmental diagnostics
**Blockchain Integration for Secure Data Management**	Decentralized Storage and Privacy	Secure peer-to-peer data sharing; avoids centralized servers	Secure real-time diagnostics and telemedicine applications
**Blockchain Integration for Secure Data Management**	AI and Blockchain Synergy	Encrypted data processed by AI; predictive analytics	Secure and intelligent chronic disease management
**Self-Powered, Bioengineered, and Autonomous Sensors**	Perovskite Solar-Powered Wearables	Battery-free; monitors glucose, pH, sweat rate, temperature; high efficiency	Continuous wearable biosensing for personalized medicine
**Self-Powered, Bioengineered, and Autonomous Sensors**	Dye-Sensitized Solar Cells (DSSCs)	Low-power; uses color changes for detection; effective in low-light environments	Non-invasive detection of prostate cancer biomarker (sarcosine)
**Self-Powered, Bioengineered, and Autonomous Sensors**	Enzymatic and Mechanical Energy Harvesting	Biofuel cells, triboelectric nanogenerators (TENGs), and piezoelectric materials as power sources	Sustainable energy for wearable and implantable biosensors

## Data Availability

This article is a review and does not report new experimental data. All information is based on previously published studies, which have been appropriately cited.
